# Evaluation of transmission-blocking potential of PvPSOP25 using transgenic murine malaria parasite and clinical isolates

**DOI:** 10.1371/journal.pntd.0012231

**Published:** 2024-06-12

**Authors:** Biying Zhang, Hao Feng, Yan Zhao, Di Zhang, Xinxin Yu, Yusi Li, Ying Zeng, Sataporn Thongpoon, Wanlapa Roobsoong, Yudi Wu, Fei Liu, Jetsumon Sattabongkot, Hui Min, Liwang Cui, Yaming Cao

**Affiliations:** 1 Department of Immunology, College of Basic Medical Sciences, China Medical University, Shenyang, Liaoning, China; 2 Department of Ophthalmology, The First Affiliated Hospital of China Medical University, Shenyang, Liaoning, China; 3 Mahidol Vivax Research Unit, Faculty of Tropical Medicine, Mahidol University, Thailand; 4 Department of Internal Medicine, Morsani College of Medicine, University of South Florida, Tampa, Florida, United States of America; Cornell University, UNITED STATES

## Abstract

**Background:**

Malaria transmission-blocking vaccines (TBVs) aim to inhibit malaria parasite development in mosquitoes and prevent further transmission to the human host. The putative-secreted ookinete protein 25 (PSOP25), highly conserved in *Plasmodium* spp., is a promising TBV target. Here, we investigated PvPSOP25 from *P*. *vivax* as a TBV candidate using transgenic murine parasite *P*. *berghei* and clinical *P*. *vivax* isolates.

**Methods and findings:**

A transgenic *P*. *berghei* line expressing PvPSOP25 (*TrPvPSOP25Pb*) was generated. Full-length PvPSOP25 was expressed in the yeast *Pichia pastoris* and used to immunize mice to obtain anti-rPvPSOP25 sera. The transmission-blocking activity of the anti-rPvPSOP25 sera was evaluated through *in vitro* assays and mosquito-feeding experiments. The antisera generated by immunization with rPvPSOP25 specifically recognized the native PvPSOP25 antigen expressed in *TrPvPSOP25Pb* ookinetes. *In vitro* assays showed that the immune sera significantly inhibited exflagellation and ookinete formation of the TrPvPSOP25Pb parasite. Mosquitoes feeding on mice infected with the transgenic parasite and passively transferred with the anti-rPvPSOP25 sera showed a 70.7% reduction in oocyst density compared to the control group. In a direct membrane feeding assay conducted with five clinical *P*. *vivax* isolates, the mouse anti-rPvPSOP25 antibodies significantly reduced the oocyst density while showing a negligible influence on mosquito infection prevalence.

**Conclusions:**

This study supported the feasibility of transgenic murine malaria parasites expressing *P*. *vivax* antigens as a useful tool for evaluating *P*. *vivax* TBV candidates. Meanwhile, the moderate transmission-reducing activity of the generated anti-rPvPSOP25 sera necessitates further research to optimize its efficacy.

## Author summary

The persistence of *Plasmodium vivax* poses a significant public health concern in certain regions, particularly Southeast Asia. The distinctive biology of *Plasmodium vivax* presents challenges for its control and eradication efforts. The development of a transmission-blocking vaccine is considered an essential strategy for malaria elimination, while the identification of antigen candidates plays a critical role in vaccine development. In this study, we aimed to evaluate the potential of PvPSOP25 as a transmission-blocking vaccine candidate using the transgenic murine parasite *P*. *berghei*. The transmission-reducing activity of anti-rPvPSOP25 sera was evaluated by both *in vitro* and mosquito-feeding experiments. Additionally, a direct membrane feeding assay using clinical *P*. *vivax* isolates from Thailand further validated the moderate transmission-reducing activity exhibited by the anti-rPvPSOP25 antisera.

## Introduction

Malaria, a parasitic disease infected by *Plasmodium* parasites transmitted by mosquitoes, poses a major threat to global health. In 2022, there were 249 million malaria cases and 608,000 deaths worldwide [1]. Among the five *Plasmodium* spp. that infect humans, *P*. *vivax* is the most geographically widespread species and responsible for most cases outside Africa. Moreover, the control and elimination of *P*. *vivax* malaria represent a great challenge due to the unique biology of this species [2]. The major obstacle in cultivating *P*. *vivax* is its preference for invading reticulocytes. *P*. *vivax* has faster sexual development, which enables it to be transmitted to mosquito vectors before the appearance of symptoms. The formation of hypnozoites in the human liver is responsible for relapses, which require the use of 8-aminoquinoline drugs for radical cures. In addition, there is a large reservoir of asymptomatic *P*. *vivax* infections in low-transmission areas that sustain transmission and hinder malaria elimination [3,4]. Moreover, the emergence of parasite drug resistance adds more difficulties to managing *P*. *vivax* malaria [5].

Vaccination is the most successful approach to controlling or eliminating infectious diseases. Unfortunately, the vaccine development for *P*. *vivax* lags behind that of *P*. *falciparum*, with a limited number of *P*. *vivax* vaccines undergoing clinical trials [6]. The delay in vaccine development is partially attributed to the significant knowledge gap regarding the biology of *P*. *vivax*. Generally, malaria vaccines are categorized as pre-erythrocytic stage, blood-stage, and transmission-blocking (TB) vaccines (TBVs). TBVs aim to reduce transmission and eliminate malaria through herd immunity [7]. The basic principle of a TBV is to immunize individuals with sexual-stage antigens of the malaria parasite or mosquito antigens to induce the production of specific antibodies, thus preventing subsequent development of the parasite in the mosquito and cutting off its transmission [8–10]. The early and continuous production of gametocytes during *P*. *vivax* infections suggests that TBV is a promising strategy for eliminating *P*. *vivax*.

Currently, only a limited number of leading *P*. *vivax* TBV candidates are in Phase I of the vaccine development pipeline [11]. The first step and the key challenge for developing a vaccine are the discovery of promising candidates. The inability to establish a long-term *in vitro* culture of *P*. *vivax* has slowed down antigen discovery for this parasite. Until now, the effective method for identifying potential candidates for *P*. *vivax* vaccines has been through studies of naturally acquired immunity in endemic populations [12] or *in silico* analysis and computational screening [13]. Besides, chimeric *P*. *knowlesi* or *P*. *berghei* lines were successfully used to exchange candidate genes with their *P*. *vivax* orthologs and to evaluate *P*. *vivax* vaccine targets [6,14].

In a previous study, we found that putative secreted ookinete protein PbPSOP25 (PBANKA_1119200) had notable transmission-blocking activity (TBA) in the P. *berghei* rodent malaria model [15]. Both polyclonal and monoclonal anti-PSOP25 antibodies can significantly reduce the formation of ookinetes *in vitro* and decrease the mosquito infection rate and oocyst density in the midgut [15]. Another group found that PIMMS43 (synonym of PSOP25) was also essential for sporogonic development in the oocyst. Moreover, antibodies against PIMMS43 interfered with parasite immune evasion during the life cycle in the mosquito, supporting it as a potential candidate for TBVs [16]. Here, we studied the transmission-blocking effect of the *P*. *vivax* ortholog PvPSOP25 (PVX_114125) using a transgenic rodent parasite model expressing the *P*. *vivax* protein and *P*. *vivax* clinical isolates. We confirmed that PvPSOP25 is located on the plasma membrane of ookinetes in the transgenic parasite line. The antibody transfer experiment and direct membrane feeding assays (DMFA) prove that the anti-rPvPSOP25 sera have moderate transmission-reducing activity (TRA).

## Materials and methods

### Ethics statement

Animal experiments were performed in accordance with the guidelines established by China Medical University animal facilities central (permit No. CMU2022457). The mice were maintained under “specific pathogen-free” conditions, and kept at a room temperature under a 12 h light/dark cycle.

### Mice, parasites, and mosquitoes

Six to eight-week-old female BALB/c mice purchased from Beijing Animal Institute were used in the animal experiments. The *P*. *berghei* ANKA strain 2.34 and *ΔPbpsop25* parasites were maintained in mice and used for challenge infection as described previously [15]. *Anopheles stephensi* mosquitoes (Hor strain) were fed 10% (w/v) glucose solution and placed in an insectary under 25 °C and 50–80% relative humidity. All animal experiments were carried out following the guidelines of the animal ethics committee of China Medical University.

### Expression of recombinant PvPSOP25 (rPvPSOP25) and immunization

The full-length rPvPSOP25 was expressed in the yeast *Pichia pastoris*. Briefly, the *Pvpsop25* DNA fragment was synthesized (GenScript Biotech Corporation) and cloned into the pPIC3.5K (+His) plasmid. A positive yeast strain was cultured in 1 L of BMG medium, and rPvPSOP25 expression was induced with methanol. Yeast cells were collected by centrifugation and lysed using an ATS high-pressure homogenizer (ATS Engineering Inc.). Recombinant proteins were purified with the Ni-NTA column (Novagen). The purified protein was analyzed using 10% SDS-PAGE and quantified by a BCA Protein Assay Kit (Beyotime).

To obtain polyclonal antibodies against rPvPSOP25, each of the ten female mice was subcutaneously immunized with 50 μg purified protein/100 μl of phosphate-buffered saline (PBS) combined with an equal volume of complete Freund’s adjuvant. Two booster immunizations with 25 μg rPvPSOP25/100 μl PBS in an equal volume of incomplete Freund’s adjuvant were performed at 14 and 28 days. The serum before immunization was used as a negative control. Antisera were collected 14 days after the last immunization and purified with Protein A columns.

### Enzyme-linked immunosorbent assay (ELISA)

Antibody titers for the anti-rPvPSOP25 sera were determined by ELISA as previously described [17]. The serum from five mice was pooled together to determine the antibody titer. In brief, the 96-well plates were coated with purified rPvPSOP25 (5 μg/ml) in a sodium carbonate buffer (0.05 M, pH 9.6) at 4 °C overnight. Plates were washed with PBST (0.05% Tween-20 in 0.1 M PBS, pH 7.4) and blocked with 1% bovine serum albumin (BSA, Sigma) at 37 °C for 1 h. The sera were diluted from 1:1000 to 1:512000 in PBS containing 1% BSA and incubated at 37 °C for 2 h. Plates were washed three times with PBST, and HRP-conjugated goat anti-mouse IgG antibodies (1:5000, Invitrogen) were added and incubated at 37 °C for 1 h. After six additional washes, 100 μl of tetramethyl benzidine (Amresco) were added and incubated in the dark for 10 min. The reaction was stopped by adding 1 mM H_2_SO_4_ and the absorbance at 490 nm was measured immediately. The value for the final dilution of the antisera was defined as that above the cut-off value of the control antisera + 3×standard deviation (SD).

### Generation of transgenic *P*. *berghei* expressing PvPSOP25 protein

**Fig 1:**
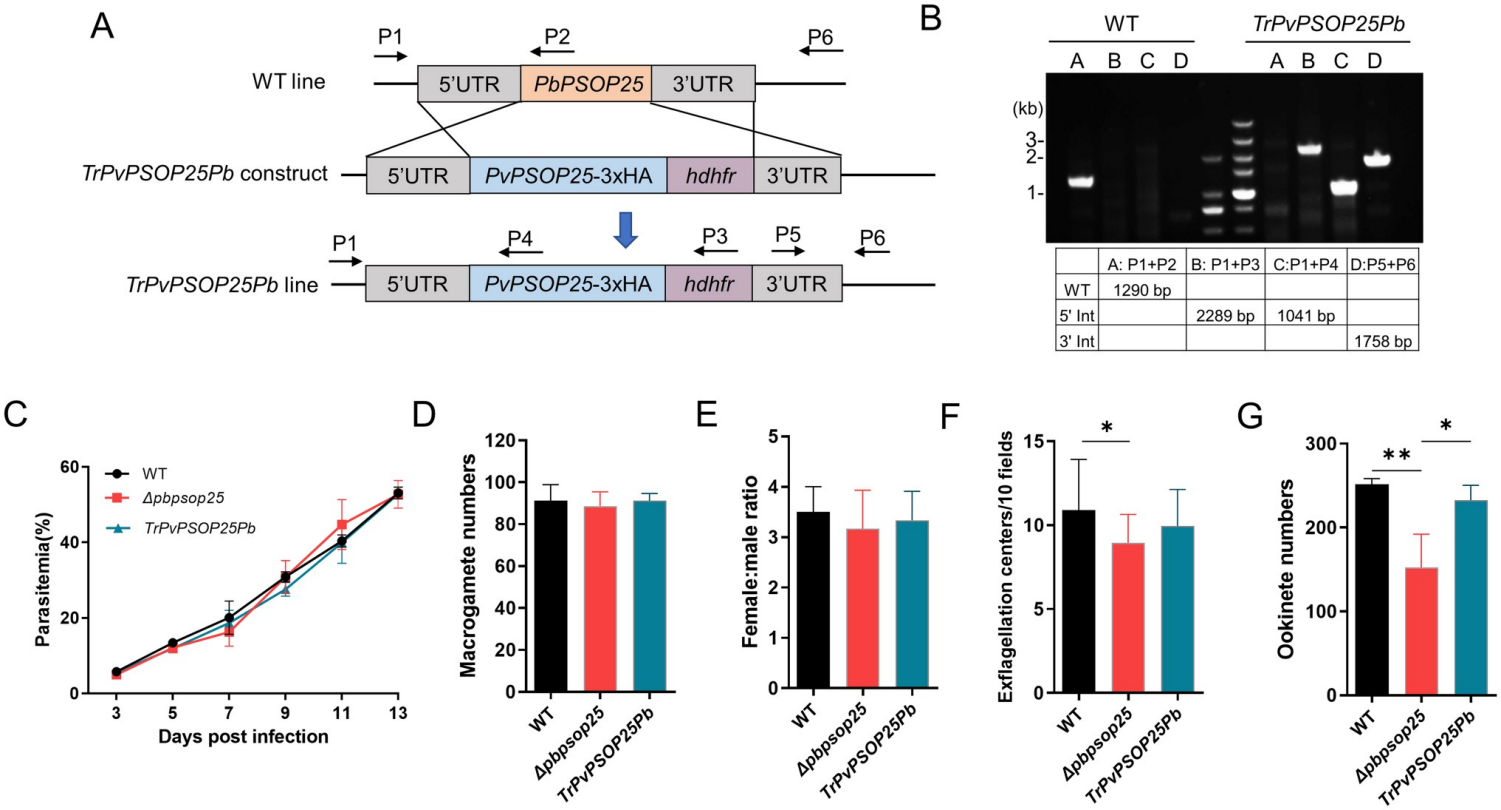
Generation and phenotypic analysis of *TrPvPSOP25Pb* parasites. (A) Schematic illustration of the generation of transgenic parasite expression *Pvpsop25* by replacing the *Pbposp25* gene with the *Pvpsop25*-3×HA and the *hdhfr* cassette. Primers 1–6 used for diagnostic PCR of the WT locus or transgenic strain are marked. (B) The positive *TrPvPSOP25Pb* parasite line was confirmed with PCR analysis using specific primers. (C) Parasitemia was determined by Giemsa-staining after infecting the mice with the WT parasite, *ΔPbpsop25*, or *TrPvPSOP25Pb* parasites, respectively. (D) After gametocyte activation, 0.5 μl of mixed cultures were used to make smears. Smears were stained with the P47 antibody for macrogametes. (E) Female/male gametocyte ratios. (F) Exflagellation centers/10 fields at 400× magnification. (G) The ookinete numbers in 0.5 μl of the *in vitro* culture were quantified through immunostaining with the anti-Pbs21 mAb. All experiments were performed in triplicates. The raw data are provided in S2 Table. Error bars indicate mean ± SD. *P < 0.05, **P < 0.01 (one way ANOVA).

The plasmid pL0034 was used to generate the *Pbpsop25* knockout strain (*ΔPbpsop25*) in our previous study [15]. The transgenic *P*. *berghei* expressing full-length *Pvpsop25* (without stop codon) in frame with a 3×HA cassette (*TrPvPSOP25Pb*) was obtained using the double-crossover homologous recombination strategy (Fig 1A). The PbPSOP25 5’ and 3’ flanking regions were amplified using primer pairs *ΔPbpsop25*-5’UTR-F-*ΔPbpsop25*-5’UTR-R and *ΔPbpsop25*-3’UTR-F-*ΔPbpsop25*-3’UTR-R, respectively (S1 Table), and cloned into the vector to flank the human *dhfr* expression cassette. The full-length *Pvpsop25* fragment with 3×*ha* tag was synthesized by GenScript Biotech Corporation and cloned into the above plasmid at the *Pst*I site adjacent to the PbPSOP25 5’UTR. Plasmid (20 μg) was linearized by *Apa*I and *Not*I digestion and electroporated into purified *P*. *berghei* schizonts using the Nucleofector system. After transfection, parasites were mixed with 100 μl of complete culture medium, and the mixture was inoculated into mice. Twenty-four hours later, parasites were selected with pyrimethamine (70 μg/ml) via drinking water for mice. Clones were then obtained by limiting dilution. Parasite genomic DNA was extracted from infected blood to confirm the *PvPSOP25* gene deletion using integration-specific PCR with primer pairs P1+P2, P1+P3, P1+P4, and P5+P6 (S1 Table).

### Phenotypic analysis of the TrPvPSOP25Pb parasites

Three female BALB/c mice in each group were infected via the tail vein with 5×10^6^ infect red blood cells (RBCs) with the wild-type (WT) *P*. *berghei*, *ΔPbpsop25*, and the *TrPvPSOP25Pb* parasite, respectively. Giemsa-stained thin blood films were used to monitor parasitemia and gametocyte sex ratio (female/male ratio). Tail blood samples of 10 μl per mouse were taken and mixed with 40 μl culture medium. The mixture was then activated at 25 °C for 30 min. After adding 50 μl culture medium, a volume of 0.5 μl of mixed cultures was utilized to prepare smears, which were subsequently fixed and probed with the anti-P47 antibody (1:1000) and Alexa Fluor 488-conjugated goat anti-rabbit IgG antibodies (1:1000). The number of macrogametes was quantified using an Olympus fluorescence microscope. The number of exflagellation centers and ookinetes were qualified, as reported previously [18]. Briefly, 10 μl of infected blood were added to 40 μl ookinete culture medium (RPMI 1640 containing 50 mg/l penicillin, 50 mg/l streptomycin, 100 mg/l neomycin, 25% (v/v) fetal bovine serum, and 6 U/ml heparin, pH 8.3) and incubated at 25 °C for 15 min. Then, the culture was spotted onto a multi-well slide. The number of exflagellation centers in 10 view fields (six wells were determined per mouse) was quantified using a phase-contrast microscope at 400× magnification. To count ookinetes, 10 μl of infected blood were added to 90 μl of ookinete culture medium and incubated at 19 °C for 24 h. Then, 0.5 μl of the culture were labeled with the anti-Pbs21 sera (for an ookinete marker) (1:500), and ookinete numbers were counted under a fluorescence microscope (100× oil objective).

### Western blot

The purification of ookinetes was performed as previously described [15]. Purified parasite antigens (10 μg) were separated by 10% SDS-PAGE under reducing conditions. Proteins were transferred to PVDF membranes (Bio-Rad), blocked with 5% non-fat milk in TBST (Tris-buffered saline with 0.1% Tween 20) at room temperature for 2 h, and then incubated with the anti-rPvPSOP25 sera (1:200) or anti-HA mAb (1:1000, Invitrogen). The anti-HSP70 serum (1:1000) was used as a loading control. The membrane was washed three times with TBST and incubated with HRP-conjugated anti-mouse antibodies (1:5000, Invitrogen). The blot was developed using an ECL Western Blotting Kit (Thermo Fisher Scientific).

### Indirect immunofluorescence assay (IFA)

The localization of PvPSOP25 in the ookinete stage was determined by IFA. Parasites were fixed with 4% paraformaldehyde and 0.0075% glutaraldehyde in PBS for 30 min at room temperature. After washing once with PBS, parasites were treated with 0.1% Triton X-100 for 10 min and blocked with 3% BSA for 1 h. After the wash, the mouse anti-rPvPSOP25 sera or anti-HA mAb (1:200, Invitrogen) were added, and samples were incubated for 1 h at 37°C. Ookinetes were co-incubated with rabbit antisera against Pbs25 (1:200). Slides were washed three times and probed with Alexa Fluor 488-conjugated goat anti-mouse IgG antibodies (1:500, Invitrogen) and Alexa Fluor 555-conjugated goat anti-rabbit IgG antibodies (1:500, Abcam) for 1 h at 37 °C. The nucleus was stained with Hoechst 33258 (1:1000, Invitrogen). Images were captured and processed on a Zeiss Axio Observer Z2 using Axio Vision software.

### *In vitro* and *in vivo* quantification of TB activities

An *in vitro* assay was carried out to determine the TBA of the immune sera on exflagellation and ookinete formation. Ookinete cultures of the WT and *TrPvPSOP25Pb* parasites were set up as described above with the culture medium containing the anti-rPvPSOP25 sera at final dilutions of 1:5, 1:10, and 1:50. Exflagellation centers were counted at 15 min post activation, and ookinete numbers were estimated after incubating at 19 °C for 24 h, as described above.

For the antibody transfer experiment, three mice were injected intravenously with 100 μl of anti-rPvPSOP25 sera 1 h before mosquito feeding. Four-day-old female *An*. *stephensi* mosquitoes, starved for 24 h, were allowed to feed on antibody-transferred mice for 30 min. Engorged mosquitoes were maintained in an insectary at 19 °C and 70% relative humidity. Ten days after feeding, 30 mosquitoes were dissected, and midguts were stained with 0.5% mercurochrome (Sigma-Aldrich) to count the number of oocysts per midgut [19].

### Direct membrane feeding assay (DMFA)

Patients visiting a malaria clinic at the Thai-Myanmar border were diagnosed with *P*. *vivax* infection by microscopy and later confirmed by PCR. Parasite densities in blood samples were quantified based on the number of parasites/200 leucocytes on thick blood smears. The five *P*. *vivax* cases had asexual parasite densities of 6600, 11200, 25760, 13400, and 4320 parasites/μL and gametocyte densities of 1000, 600, 2920, 1360 and 1200 gametocytes/μL, respectively. Written informed consent was obtained from these volunteers before drawing 5 mL of venous blood. DMFA was performed using a published protocol [20]. Briefly, purified antibodies against rPvPSOP25 or pre-immune sera were diluted with normal human AB+ serum at the ratio of 1:1 in a total volume of 180 μl, which were mixed with RBCs from *P*. *vivax* patients (1:1, v/v). Each reconstituted blood sample was introduced into a membrane feeder after incubation at 37 °C for 15 min. Female *Anopheles dirus* mosquitoes were allowed to feed on the reconstituted blood for 30 min. After removing unfed mosquitoes, the remaining mosquitoes were maintained in an insectary for 7 days. Twenty mosquitoes from each group were dissected, and oocysts in the midguts were counted by microscopy.

### Analysis of genetic polymorphisms

Genomic DNA from dried filter-paper blood spots of *P*. *vivax* isolates used for the DMFA was extracted using a QIAamp DNA Blood Mini kit (Qiagen). The 705 bp *Pvpsop25* DNA fragment was amplified by PCR with primers (PVX_114125-F and PVX_114125-R) designed based on the Sal-I sequence. PCR fragments were analyzed by electrophoresis on a 1% agarose gel. The purified PCR products were sequenced using the ABI Prism BigDye cycle sequencing kit (Applied Biosystems).

### Statistical analysis

Statistical analysis was carried out using SPSS software, version 22.0. Antibody titers were compared using the Student’s *t*-test. Parasitemia, macrogamete numbers, exflagellation centers, and ookinete numbers among groups were compared by one-way ANOVA. The *in vitro* inhibition assays were analyzed by two-way ANOVA followed by Tukey’s multiple comparison test. The prevalence of infection was analyzed using Fisher’s exact test, while the intensity of infection (oocysts/midgut) was measured using the Mann-Whitney *U* test. *P* < 0.05 was considered statistically significant.

## Results

### Transgenic *P*. *berghei* parasite line expressing PvPSOP25

To generate the chimeric *TrPvPSOP25Pb* parasite line, we replaced the *Pbpsop25* gene (PBANKA_1119200) with the *Pvpsop25* gene (PVX_114125) in *P*. *berghei* (Fig 1A). The transgenic *TrPvPSOP25Pb* line was selected with pyrimethamine, and integration-specific PCR was performed to verify the successful genomic integration of the *Pvsop25-HA* gene (Fig 1B).

It has been demonstrated that the deletion of *Pbpsop25* contributes to a noticeable decrease in male gametocyte exflagellation and ookinete conversion rate, ultimately resulting in a reduction in midgut oocyst density in the *ΔPbpsop25* line [15]. To explore whether the *P*. *vivax* ortholog *Pvpsop25* functions similarly in the murine malaria parasite, we analyzed the phenotypes of the transgenic parasite. Similar to the *ΔPbpsop25* line, the *TrPvPSOP25Pb* line exhibited no discernible differences in parasite asexual growth (Fig 1C), the number of macrogametes (Fig 1D), and the female/male sex ratio (Fig 1E) when compared to the WT parasite. As observed previously, *ΔPbpsop25* substantially reduced the exflagellation of male gametocytes and the formation of ookinetes. However, introducing *Pvpsop25* completely restored the slight male gametocyte exflagellation defect (Fig 1F), and the number of ookinetes formed was similar to the WT (Fig 1G). These observations demonstrated the conserved function of the PSOP25 proteins in the transmission stages of *P*. *vivax* and *P*. *berghei*, supporting that *P*. *berghei* could be used as a model for screening *P*. *vivax* malaria vaccine candidates.

### Generation of antiserum against PvPSOP25 protein

**Fig 2:**
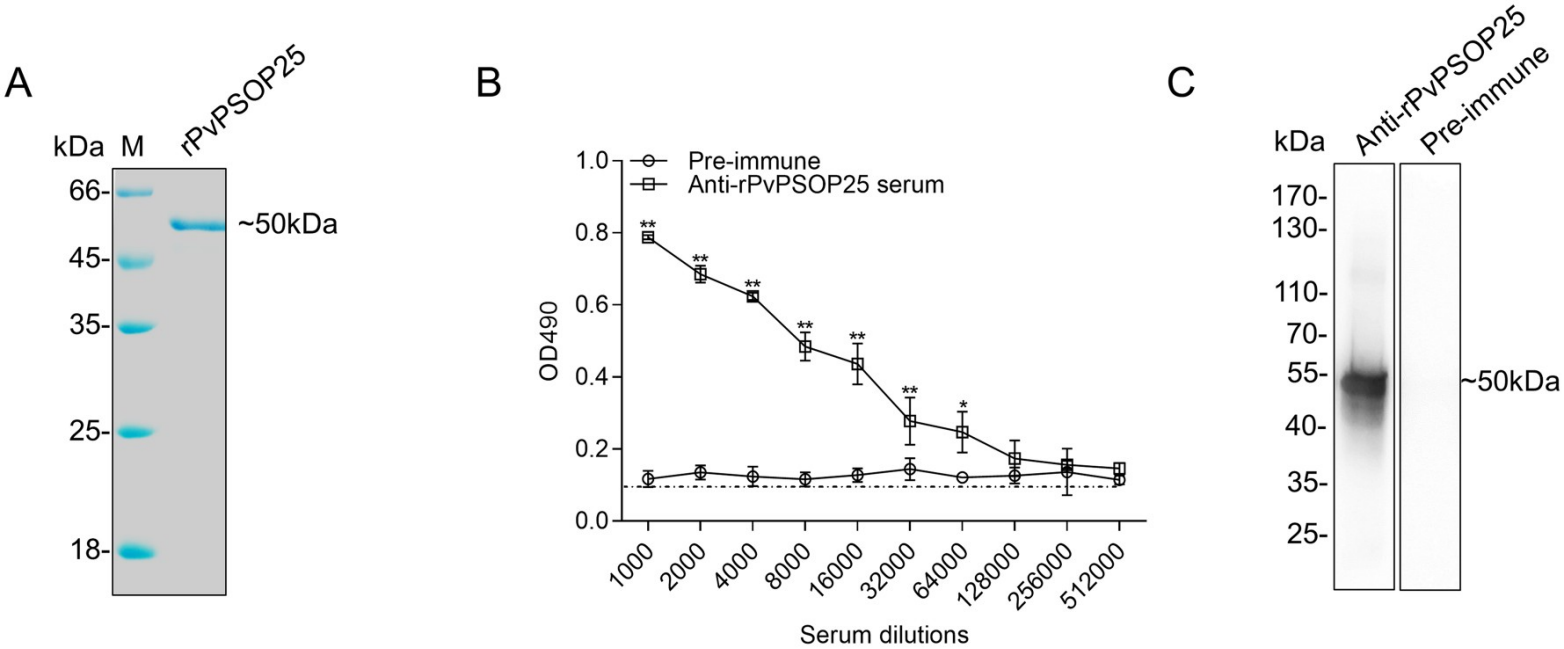
Generation of anti-rPvPSOP25 serum. (A) Purified rPvPSOP25 protein was separated on a 10% SDS-PAGE and stained with Coomassie brilliant blue. (B) Antibody titers for the elicited anti-rPvPSOP25 sera were determined by ELISA on day 14 after the final immunization. Pre-immune sera collected from the mice was used as the control. The error bar shows mean ± SD. * *P* < 0.01, ** *P* < 0.001 (Student’s *t*-test). (C) The purified rPvPSOP25 protein was subjected to Western blot analysis using the pre-immune and anti-rPvPSOP25 sera, respectively.

To determine the immunogenicity and TB potential of PvPSOP25 as a TBV candidate, we expressed the full-length PvPSOP25 protein in the yeast *Pichia pastoris* expression system (S1A Fig). The protein sequence alignment revealed only 24% identity between PvPSOP25 and PbPSOP25 (S1A and S1B Fig). The recombinant protein was purified using a Ni-NTA column and subsequently verified by SDS-PAGE analysis, showing an expected single band of ~50 kDa (Fig 2A). The purified rPvPSOP25 protein was used to immunize mice to obtain polyclonal antibodies. Serum collected after the final immunization was quantified using ELISA, revealing an antibody titer of 1:512000 (Fig 2B). Western blot was also performed with purified rPvPSOP25 protein (Fig 2C). Compared with the pre-immune sera, the immune antisera recognized the rPvPSOP25 band at approximately 50 kDa.

### The expression and localization of PvPSOP25 in transgenic parasites

**Fig 3:**
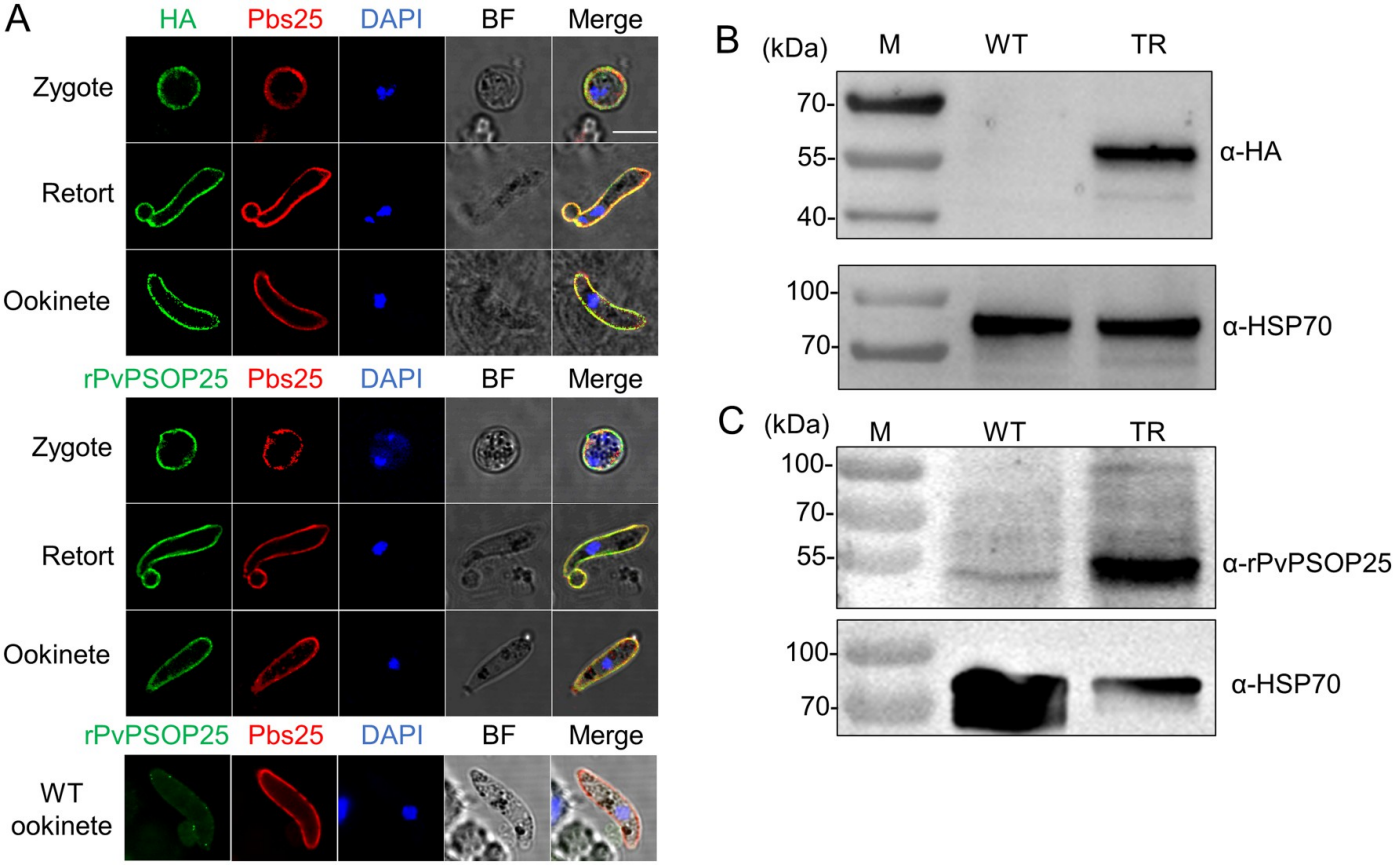
Specific recognition of PvPSOP25 by the anti-rPvPSOP25 sera. (A) IFA was performed using TrPvPSOP25Pb zygote, retort, and ookinete with either the anti-HA mAb (upper panel) or anti-rPvPSOP25 sera (middle panel). *P*. *berghei* (WT) ookinetes were immunostained with the anti-rPvPSOP25 mAb (green) (lower panel). The zygotes/retorts/ookinetes were also labeled with the anti-Pbs25 antibody. Nuclei were labeled with DAPI. BF, bright field. Scale bars, 5 μm. (B, C) Western blot analysis was performed on *P*. *berghei* (WT) and *TrPvPSOP25Pb* (TR) ookinete lysates using (B) the anti-HA mAb and (C) the anti-rPvPSOP25 sera. Anti-HSP70 sera served as loading control.

PbPSOP25 was predominately expressed on the surface of zygotes, retorts, and mature ookinetes [15,21]. To characterize the expression of PvPSOP25 in transgenic parasites, IFA was performed with zygotes, retorts, and ookinetes. Both anti-HA monoclonal antibody (mAb) and anti-rPvPSOP25 sera were used to detect the PvPSOP25 antigen in the transgenic line. A localization pattern consistent with the previous study on PbPSOP25 in WT parasites was observed [15]. The fluorescence signal showed association with the plasma membrane at these stages and co-localized with Pbs25, an ookinete surface marker. Similar results were obtained using both the anti-HA mAb and anti-rPvPSOP25 sera (Fig 3A). In contrast, anti-rPvPSOP25 sera did not react with the PbPSOP25 in *P*. *berghei*.

In parallel, we purified the ookinetes of the *TrPvPSOP25Pb* line to detect the expression of PvPSOP25 by Western blot analysis with both anti-HA mAb and anti-rPvPSOP25 sera. A specific band of ~50 kDa was detected in the TrPvPSOP25Pb ookinete lysates but not in the WT parasites (Fig 3B), which is consistent with the IFA results. These results demonstrated that PvPSOP25 showed a similar expression pattern as PbPSOP25 in *TrPvPSOP25Pb*, and the anti-rPvPSOP25 sera specifically recognized PvPSOP25 but not PbPSOP25.

### Evaluation of TBA of the anti-rPvPSOP25 sera

**Fig 4:**
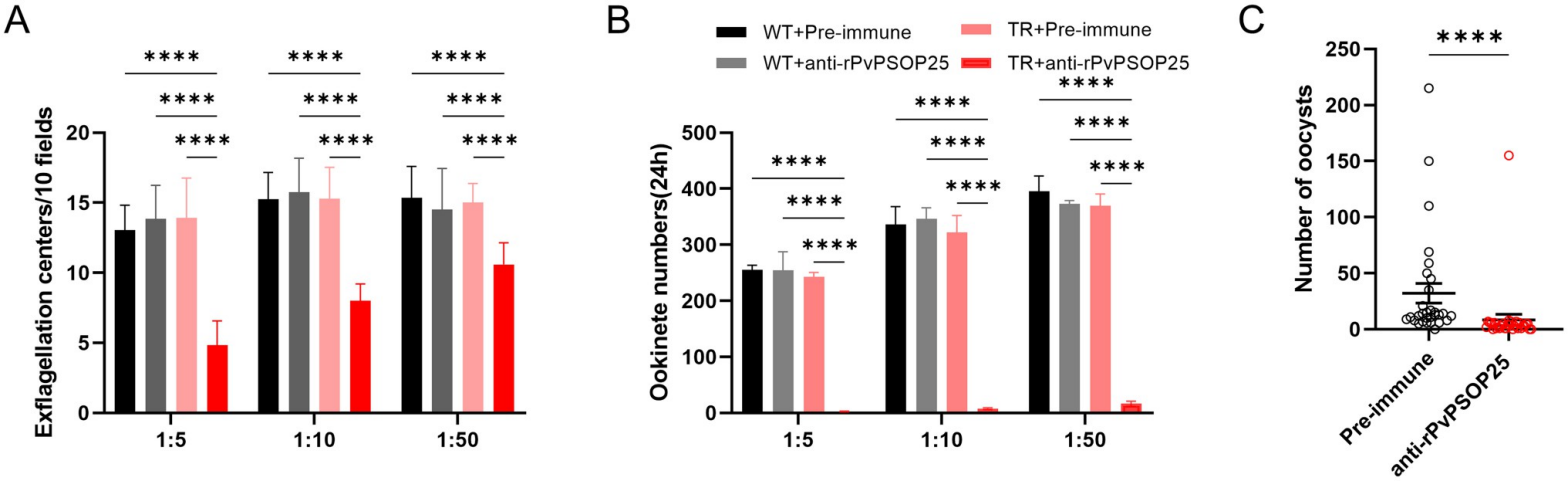
Evaluation of TBA of anti-rPvPSOP25 sera by *in vitro* and *in vivo* assays. The inhibition of the anti-rPvPSOP25 sera on (A) male gametocyte exflagellation and (B) ookinete formation was assessed by *in vitro* assays. The pre-immune and anti-rPvPSOP25 sera were diluted at 1:5, 1:10, and 1:50 and incubated with the WT *P*. *berghei* or the *TrPvPSOP25Pb* parasites (TR). Data were representative of three separate experiments. The error bar shows mean ± SD. ****P < 0.0001 (Two-way ANOVA followed by Tukey’s multiple comparison test). (C) Mice infected with the *TrPvPSOP25Pb* parasites were passively transferred with pre-immune or anti-rPvPSOP25 sera and subjected to the mosquito-feeding assay. Oocyst numbers per midgut were counted 10 days after mosquito feeding. Data of mosquito feeding assays are shown in Table 1. Error bars indicate mean ± SEM. **** *P* < 0.0001 (Mann-Whitney *U* test).

Next, we evaluated the activities of the anti-PvPSOP25 sera for inhibiting male gametocyte exflagellation and ookinete formation using *in vitro* assays. Anti-rPvPSOP25 sera were diluted with the medium at 1:5, 1:10, and 1:50, respectively, and subsequently incubated with *P*. *berghei* WT or the *TrPvPSOP25Pb* parasites. We observed a negligible impact of the anti-rPvPSOP25 sera on male exflagellation (Fig 4A) and ookinete formation (Fig 4B) of the WT *P*. *berghei* parasites, consistent with the sequence divergence between PvPSOP25 and PbPSOP25. When the *TrPvPSOP25Pb* parasites were incubated with the diluted anti-rPvPSOP25 sera, the number of exflagellation centers was reduced by 66%, 48%, and 29%, respectively, compared to the pre-immune control group (*P* < 0.0001, Fig 4A). With the increase in the dilution ratio, the inhibitory effect of the anti-rPvPSOP25 antibodies on exflagellation was weakened. Significantly, following incubation with the parasite in the diluted medium for 24 h, a remarkable reduction of 99% (1:5), 98% (1:10), and 95% (1:50) in the number of ookinetes formed was observed compared to the pre-immune group (*P* < 0.0001, Fig 4B).

To further assess the TRA and TBA of the antisera *in vivo*, mice infected with the TrPvPSOP25Pb parasite were passively transferred with either the pre-immune or anti-PvPSOP25 sera through the tail veins and subjected to the mosquito feeding assay. At 10 days post-feeding, 30 mosquitoes were dissected from each feeding group to determine midgut oocyst density and the prevalence of infection. The mean number of oocysts per midgut in mosquitoes feeding on mice receiving the anti-rPvPSOP25 sera was 9.7, which was significantly lower than the pre-immune serum group (33.3) (*P* < 0.0001, Mann-Whitney *U* test; Fig 4C). This reflected a 70.7% reduction in the mean oocyst density. In comparison, the anti-PvPSOP25 sera only resulted in a 10.1% reduction in infection prevalence compared to the control group (Table 1).

**Table 1:** *In vivo* evaluation of transmission-blocking effects of PvPSOP25 in mosquito feeding experiments.

Group	Oocyst density mean (range) (n = 30)	TRA ^a^	Prevalence of infection mean (n = 30)	TBA ^b^
Pre-immune	33.3 (0–215)		96.8 (29/30)	
anti-rPvPSOP25	9.7 (0–155)	70.7%	86.7 (26/30)	10.1%

^a^ TRA was calculated as (mean oocyst density _Pre-immune_—mean oocyst density _anti-rPvPSOP25_)/mean oocyst density _Pre-immune_ × 100%

^b^ TBA was calculated as % prevalence _Pre-immune_—% prevalence _anti-rPvPSOP25_

### Evaluation of PvPSOP25’s TB potential using DMFA

**Fig 5:**
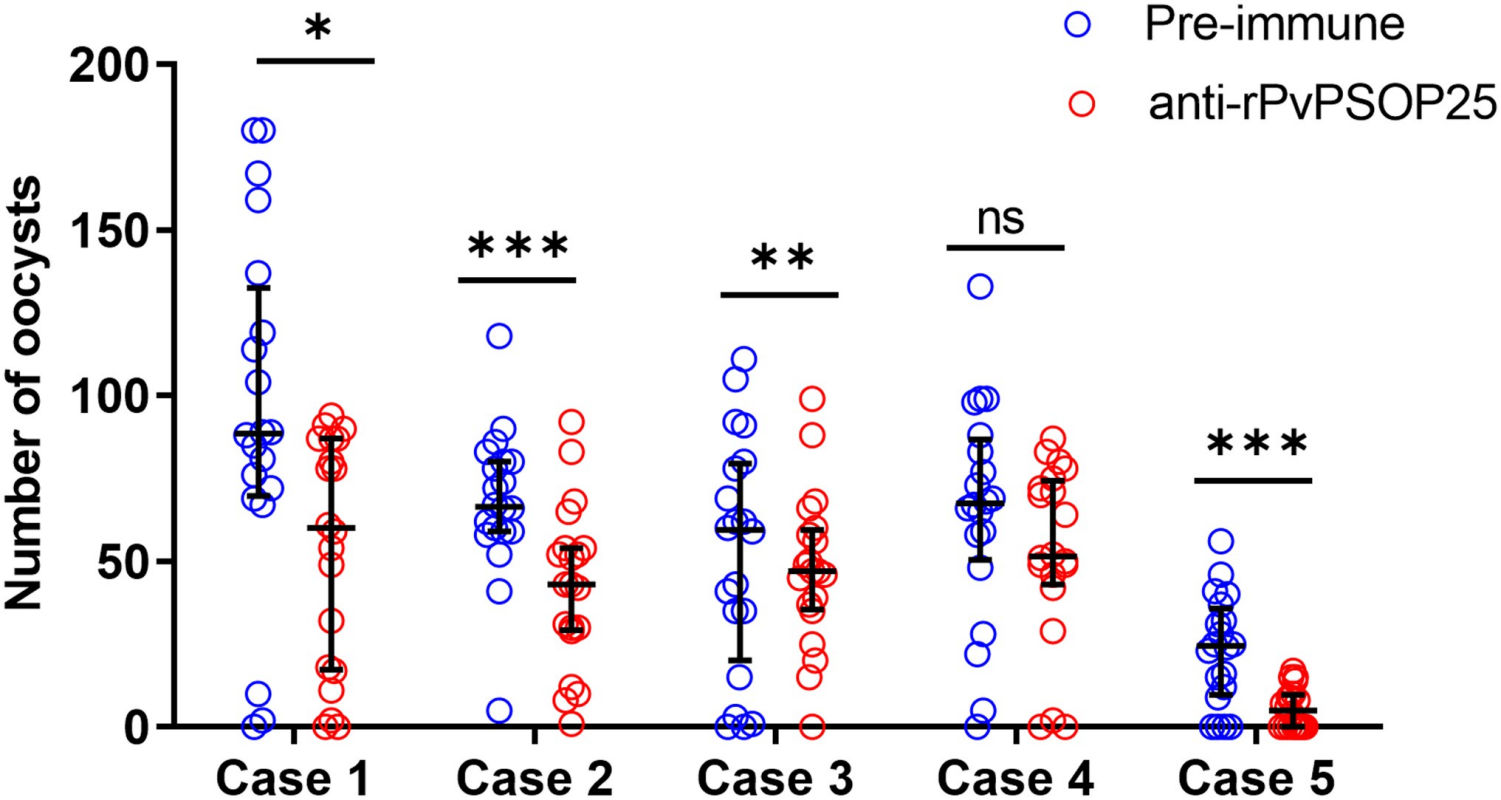
Evaluation of the transmission-reducing activity of anti-rPvPSOP25 sera by DMFA. DMFA was carried out using five *P*. *vivax* isolates with anti-rPvPSOP25 or pre-immune sera from mice mixed with heated-inactive (complement minus) AB^+^ human serum in the ratio of 1:1. Scattered dots represent the number of oocysts in mosquito midguts. The long horizontal bar represents the median number of oocysts in each group, and the two short horizontal bars represent the interquartile ranges. * *P* < 0.05, **P < 0.01, ***P < 0.001 (Mann-Whitney *U* test).

Subsequently, five clinical isolates of *P*. *vivax* samples were used to investigate the TB potential of PvPSOP25 by DMFA. Although the anti-rPvPSOP25 sera did not significantly reduce the prevalence of mosquito infection (Table 2), they possessed evident TRA. For the four field isolates (case 1, 2, 3, and 5), the mean oocyst densities in mosquitoes fed with the anti-rPvPSOP25 sera were decreased by 43.0, 37.3, 57.0 and 75.2%, respectively, compared to the pre-immune control (*P* < 0.05, Fig 5 and Table 2).

**Table 2:** Transmission-blocking effect of anti-rPvPSOP25 sera with five clinical *P*. *vivax* isolates.

*P*. *vivax* isolates	Antibodies	Oocyst number Median (IQR) ^a^	Mean oocysts number	% inhibition of oocyst^b^	*P* value 1^c^	Infection rate (%) ^d^	% inhibition of prevalence^e^	*P* value 2^f^
Case 1	Pre-immu	88.5 (69.8–132.5)	94.4			95 (19/20)		
	rPvPSOP25	60.0 (17.3–87.0)	53.8	43.0	0.0145	90 (18/20)	5.3	1.000
Case 2	Pre-immu	66.5 (59.0–80.0)	67.8			100 (20/20)		
	rPvPSOP25	43.0 (29.3–54.0)	42.5	37.3	0.0009	100 (20/20)	0.0	1.000
Case 3	Pre-immu	59.5 (20.0–79.5)	52.1			90 (18/20)		
	rPvPSOP25	24.0 (10.8–36.3)	22.4	57.0	0.0066	100 (20/20)	-	-
Case 4	Pre-immu	67.5 (50.5–86.8)	65.3			95 (19/20)		
	rPvPSOP25	51.5 (43.0–74.3)	52.5	19.6	0.2035	95 (19/20)	0.0	1.000
Case 5	Pre-immu	24.5 (9.8–35.8)	23.0			80 (16/20)		
	rPvPSOP25	5.0 (0.0–9.8)	5.70	75.2	0.0004	80 (16/20)	0.0	1.000

^a^ IQR: inter-quartile range.

^b^ % inhibition of oocyst intensity was calculated as (mean control—mean test)/mean control × 100%.

^c^ Mean number of oocysts was statistically analyzed (Mann-Whitney *U* test), and *P* values less than 0.05 were considered statistically significant.

^d^ The infection prevalence was calculated by the number of oocyst-infected mosquitoes per 20 mosquitoes dissected in each group (Inf/Diss).

^e^ % inhibition of prevalence was calculated as % infection rate control—% infection rate test.

^f^ Prevalence was analyzed using Fisher’s exact test. *P* values less than 0.05 were considered statistically significant.

To investigate whether the variation in TRA among the different isolates might be attributed to genetic polymorphisms of the *psop25* gene, we sequenced the *psop25* gene from the five *P*. *vivax* isolates used in DMFA. Yet, these samples had amino acid sequences identical to that of the Sal-I strain (S2 Fig).

## Discussion

TBVs against *P*. *vivax* could be a vital component for the elimination of this parasite. A comprehensive understanding of the biology of *P*. *vivax* is critical to developing potential vaccines. TBV candidate antigens are not strongly affected by the selection pressure from the vertebrate immune system and usually show low-level polymorphism [22]. Currently, recognized candidate antigens with excellent TBA include gametocyte and gamete surface antigens P230 and P48/45 [23,24], Pfs47 [25], HAP2 [26], zygote and ookinete surface antigens P25 [27] and P28 [28], and midgut protein AnAPN1 [29]. Yet, only a limited number of candidates exist in the vaccine development pipeline for *P*. *vivax* [30]. Thus, there is a high priority to identify and expand the TBV candidate repertoire for this parasite.

Our efforts to discover TBV candidates using the rodent malaria parasite model led to the identification of PbPSOP25, which showed 31.2% TBA and 66.3% TRA in mosquito-feeding assays [15]. *Pbpsop25* plays a critical role in male gametocyte biology since its deletion led to reduced formation of exflagellation centers and subsequent fertilization defects [15]. This has prompted us to explore its suitability as a TBV candidate for *P*. *vivax*. *P*. *vivax* TBV evaluation requires clinical isolates due to the lack of long-term cultures for this parasite. To circumvent these difficulties, transgenic parasite models in *P*. *falciparum* [31], *P*. *knowlesi* [6], and *P*. *berghei* [14,32] expressing *P*. *vivax* antigens have been explored as alternatives for TBV evaluation. The transfection technology based on gene insertion/marker out and the CRISPR/Cas9 gene editing approach was successfully employed to generate a transgenic *P*. *berghei* line expressing a *P*. *vivax* gene, PvPSOP25. Despite the sequence divergence, we found that *Pvpsop25* was fully functional in *P*. *berghei*, as it completely rescued the sexual development defects in the *Pbpsop25* knockout parasites. Notably, the sequence divergence is reflected in that the anti-rPvPSOP25 sera failed to recognize the PbPSOP25 antigen in WT parasites by IFA and Western blot. Using this parasite, we evaluated antisera raised against the yeast-expressed rPvPOSP25 in mice, which confirmed the significant effect of the anti-rPvPSOP25 sera in reducing exflagellation and ookinete formation *in vitro* and midgut oocyst numbers *in vivo*. However, we found that the TRA of PvPSOP25 was slightly weaker than that for PbPOSP25 [15], which may be related to the lower antibody titers obtained for rPvPSOP25 than rPbPSOP25. Thus, future work to enhance the immunogenicity of the PvPSOP25 is warranted. A previous study has indicated that the conserved 6-cysteine domains in P48/45 and P47 play a pivotal role in forming a complex globular tertiary structure and require proper conformation to elicit transmission-blocking antibodies [33]. However, PbPSOP25 and PvPSOP25 lack identifiable functional domains, and no refolding step is needed for its immunogenicity, suggesting linear epitopes of PSOP25 may be sufficient for TBV development.

In this study, we adopted the yeast *P*. *pastoris* to express rPvPSOP25 and showed that the recombinant protein was highly immunogenic. Like the prokaryotic protein expression system, the yeast expression system offers similar advantages of simplicity, rapid growth, high protein yield, and low cost while preserving the advantages of a eukaryotic expression system, such as proper folding and efficient secretion of the protein product [34]. However, the undesired proteolytic degradation of heterologous proteins expressed in *P*. *pastoris* may lead to low product yield. Another unwanted feature of the yeast system is the glycosylation of expressed proteins, which may interfere with their antigenicity, since *Plasmodium* proteins are typically not glycosylated [35].

The assessment of the TBA of *Plasmodium* antigens critically relies on DMFA. TBA, which measures the prevalence of infected mosquitoes, appears more biologically important and epidemiologically relevant since mosquitoes carrying any number of parasites can potentially transmit the infection. Thus, it has been traditionally emphasized during TBV evaluation. However, TBA is less reproducible in field conditions and may impose an excessively stringent criterion for screening vaccine candidates. The importance of TRA, which measures the reduction in oocyst counts, has been increasingly recognized and incorporated into a comprehensive evaluation model of TBVs [36]. In this study, we also observed evident TRA of the anti-rPvPSOP25 sera using DMFA with clinical *P*. *vivax* isolates. Consistent with earlier studies [37], DMFA using field parasite isolates always results in considerable variations. Such variations may not be due to the sequence variations in the target genes. Several factors may have contributed to these variations, including gametocyte density, the proportion of mature gametocytes, and the male/female gametocyte ratio among field isolates. Of the five clinical *P*. *vivax* isolates used in DMFA, all showed substantial reductions in midgut oocyst densities, while four results were statistically significant. Altogether, the *in vivo* studies with the transgenic parasite in mice and *in vitro* DMFA using clinical *P*. *vivax* isolates all confirm the TB potential of PvPSOP25.

Developing a safe and efficacious vaccine against *vivax* malaria is imperative for controlling and eradicating this disease. Reverse vaccinology based on high-throughput *in silico* analyses of “omics” data is widely applied to screen potential vaccine candidates [38]. Besides, computational vaccinology may also facilitate the design of novel vaccines by *in silico* prediction of candidate antigens and immunogen design [39]. The combination of the wheat germ cell-free protein synthesis technology and *in vitro* immune screening methods can potentially enhance the development of TBVs [40]. Furthermore, the RNA vaccine technology used to combat the COVID-19 pandemic offers another valuable tool for screening and evaluating vaccine candidates. We can apply all these technological advancements in malaria TBV discovery.

## Supporting information

S1 Table Primers used in this study. (XLSX)

S2 Table The raw data are provided for Figs 1C–1G, 2B, 4A–4C and 5 respectively. (XLSX)

S1 Fig Sequence analysis of PSOP25. (A) PvPSOP25 contains a signal peptide (red) at the N-terminus low complexity region (pink) and transmembrane region (blue). Yeast cell expression shows amino acids 21–412. (B) Alignment of PSOP25 between *P*. *vivax* (Pv) and *P*. *berghei* (Pb). Amino acids are marked in black for identity and red for similarity. The sequence expressed in yeast cells is indicated.
(TIF)

S2 Fig Multiple sequence alignment of PvPSOP25 from *P*. *vivax* Sal-I strain and five *P*. *vivax* isolates used in DMFA. PSOP25 homologs from *P*. *vivax* reference strain (Sal-I) and five clinical samples were aligned. Identical amino acid was shadowed in red, while conserved was in white.
(TIF)

## References

[1] Geneva: World Health Organization. World malaria report 2023. 2023:Licence: CC BY-NC-SA 3.0 IGO.

[2] Kumari S, Sinha A. Culture and transfection: Two major bottlenecks in understanding Plasmodium vivax biology. Frontiers in microbiology. 2023;14:1144453. Epub 2023/04/21. doi: 10.3389/fmicb.2023.1144453 . 37082177 PMC10110902

[3] Sattabongkot J, Suansomjit C, Nguitragool W, Sirichaisinthop J, Warit S, Tiensuwan M, et al. Prevalence of asymptomatic Plasmodium infections with sub-microscopic parasite densities in the northwestern border of Thailand: a potential threat to malaria elimination. Malar J. 2018;17(1):329. Epub 2018/09/14. doi: 10.1186/s12936-018-2476-1 . 30208895 PMC6134695

[4] Nguitragool W, Mueller I, Kumpitak C, Saeseu T, Bantuchai S, Yorsaeng R, et al. Very high carriage of gametocytes in asymptomatic low-density Plasmodium falciparum and P. vivax infections in western Thailand. Parasites & vectors. 2017;10(1):512. Epub 2017/10/27. doi: 10.1186/s13071-017-2407-y . 29065910 PMC5655986

[5] Hanboonkunupakarn B, White NJ. Advances and roadblocks in the treatment of malaria. Br J Clin Pharmacol. 2022;88(2):374–82. Epub 2020/07/14. doi: 10.1111/bcp.14474 . 32656850 PMC9437935

[6] Ndegwa DN, Kundu P, Hostetler JB, Marin-Menendez A, Sanderson T, Mwikali K, et al. Using Plasmodium knowlesi as a model for screening Plasmodium vivax blood-stage malaria vaccine targets reveals new candidates. PLoS Pathog. 2021;17(7):e1008864. Epub 2021/07/02. doi: 10.1371/journal.ppat.1008864 . 34197567 PMC8279373

[7] Duffy PE. Current approaches to malaria vaccines. Curr Opin Microbiol. 2022;70:102227. Epub 2022/11/08. doi: 10.1016/j.mib.2022.102227 . 36343566 PMC11127243

[8] Carter R, Chen DH. Malaria transmission blocked by immunisation with gametes of the malaria parasite. Nature. 1976;263(5572):57–60. Epub 1976/09/02. doi: 10.1038/263057a0 986561

[9] Gwadz RW. Successful immunization against the sexual stages of Plasmodium gallinaceum. Science. 1976;193(4258):1150–1. Epub 1976/09/17. doi: 10.1126/science.959832 959832

[10] Ishino T, Tsuboi T. Progress toward a transmission-blocking vaccine against malaria. Lancet Infect Dis. 2018;18(9):927–8. Epub 2018/08/01. doi: 10.1016/S1473-3099(18)30358-X . 30061052

[11] De SL, Ntumngia FB, Nicholas J, Adams JH. Progress towards the development of a P. vivax vaccine. Expert review of vaccines. 2021;20(2):97–112. Epub 2021/01/23. doi: 10.1080/14760584.2021.1880898 . 33481638 PMC7994195

[12] Tebeje SK, Chali W, Hailemeskel E, Ramjith J, Gashaw A, Ashine T, et al. Naturally acquired antibodies to gametocyte antigens are associated with reduced transmission of Plasmodium vivax gametocytes to Anopheles arabiensis mosquitoes. Front Cell Infect Microbiol. 2022;12:1106369. Epub 2023/02/03. doi: 10.3389/fcimb.2022.1106369 . 36726645 PMC9885094

[13] Nanda Kumar Y, Jeyakodi G, Gunasekaran K, Jambulingam P. Computational screening and characterization of putative vaccine candidates of Plasmodium vivax. J Biomol Struct Dyn. 2016;34(8):1736–50. Epub 2015/09/05. doi: 10.1080/07391102.2015.1090344 . 26338678

[14] Bai J, Liu F, Yang F, Zhao Y, Jia X, Thongpoon S, et al. Evaluation of transmission-blocking potential of Pv22 using clinical Plasmodium vivax infections and transgenic Plasmodium berghei. Vaccine. 2023;41(2):555–63. Epub 2022/12/13. doi: 10.1016/j.vaccine.2022.11.058 . 36503858 PMC9812905

[15] Zheng W, Liu F, He Y, Liu Q, Humphreys GB, Tsuboi T, et al. Functional characterization of Plasmodium berghei PSOP25 during ookinete development and as a malaria transmission-blocking vaccine candidate. Parasites & vectors. 2017;10(1):8. Epub 2017/01/07. doi: 10.1186/s13071-016-1932-4 . 28057055 PMC5217559

[16] Ukegbu CV, Giorgalli M, Tapanelli S, Rona LDP, Jaye A, Wyer C, et al. PIMMS43 is required for malaria parasite immune evasion and sporogonic development in the mosquito vector. Proc Natl Acad Sci U S A. 2020;117(13):7363–73. Epub 2020/03/14. doi: 10.1073/pnas.1919709117 . 32165544 PMC7132314

[17] Miura K, Orcutt AC, Muratova OV, Miller LH, Saul A, Long CA. Development and characterization of a standardized ELISA including a reference serum on each plate to detect antibodies induced by experimental malaria vaccines. Vaccine. 2008;26(2):193–200. Epub 2007/12/07. doi: 10.1016/j.vaccine.2007.10.064 . 18054414 PMC2253722

[18] Liu F, Yang F, Wang Y, Hong M, Zheng W, Min H, et al. A conserved malaria parasite antigen Pb22 plays a critical role in male gametogenesis in Plasmodium berghei. Cell Microbiol. 2021;23(3):e13294. Epub 2020/11/23. doi: 10.1111/cmi.13294 . 33222390 PMC8194029

[19] Beetsma AL, van de Wiel TJ, Sauerwein RW, Eling WM. Plasmodium berghei ANKA: purification of large numbers of infectious gametocytes. Experimental parasitology. 1998;88(1):69–72. Epub 1998/03/21. doi: 10.1006/expr.1998.4203 . 9501851

[20] Sattabongkot J, Kumpitak C, Kiattibutr K. Membrane Feeding Assay to Determine the Infectiousness of Plasmodium vivax Gametocytes. Methods Mol Biol. 2015;1325:93–9. Epub 2015/10/10. doi: 10.1007/978-1-4939-2815-6_8 . 26450382

[21] Zheng W, Kou X, Du Y, Liu F, Yu C, Tsuboi T, et al. Identification of three ookinete-specific genes and evaluation of their transmission-blocking potentials in Plasmodium berghei. Vaccine. 2016;34(23):2570–8. Epub 2016/04/17. doi: 10.1016/j.vaccine.2016.04.011 . 27083421 PMC4864593

[22] Riley EM, Stewart VA. Immune mechanisms in malaria: new insights in vaccine development. Nature medicine. 2013;19(2):168–78. Epub 2013/02/08. doi: 10.1038/nm.3083 . 23389617

[23] van Dijk MR, Janse CJ, Thompson J, Waters AP, Braks JA, Dodemont HJ, et al. A central role for P48/45 in malaria parasite male gamete fertility. Cell. 2001;104(1):153–64. Epub 2001/02/13. doi: 10.1016/s0092-8674(01)00199-4 . 11163248

[24] Theisen M, Jore MM, Sauerwein R. Towards clinical development of a Pfs48/45-based transmission blocking malaria vaccine. Expert review of vaccines. 2017;16(4):329–36. Epub 2017/01/04. doi: 10.1080/14760584.2017.1276833 . 28043178

[25] Canepa GE, Molina-Cruz A, Yenkoidiok-Douti L, Calvo E, Williams AE, Burkhardt M, et al. Antibody targeting of a specific region of Pfs47 blocks Plasmodium falciparum malaria transmission. NPJ Vaccines. 2018;3:26. Epub 2018/07/14. doi: 10.1038/s41541-018-0065-5 . 30002917 PMC6039440

[26] Angrisano F, Sala KA, Da DF, Liu Y, Pei J, Grishin NV, et al. Targeting the conserved fusion loop of HAP2 inhibits the transmission of Plasmodium berghei and falciparum. Cell Rep. 2017;21(10):2868–78. Epub 2017/12/07. doi: 10.1016/j.celrep.2017.11.024 . 29212032 PMC5732318

[27] Menon V, Kapulu MC, Taylor I, Jewell K, Li Y, Hill F, et al. Assessment of antibodies induced by multivalent transmission-blocking malaria vaccines. Front Immunol. 2017;8:1998. Epub 2018/02/07. doi: 10.3389/fimmu.2017.01998 . 29403479 PMC5780346

[28] Kim TS, Kim HH, Moon SU, Lee SS, Shin EH, Oh CM, et al. The role of Pvs28 in sporozoite development in Anopheles sinensis and its longevity in BALB/c mice. Experimental parasitology. 2011;127(2):346–50. Epub 2010/08/31. doi: 10.1016/j.exppara.2010.08.015 . 20801117

[29] Atkinson SC, Armistead JS, Mathias DK, Sandeu MM, Tao D, Borhani-Dizaji N, et al. The Anopheles-midgut APN1 structure reveals a new malaria transmission-blocking vaccine epitope. Nature structural & molecular biology. 2015;22(7):532–9. Epub 2015/06/16. doi: 10.1038/nsmb.3048 . 26075520 PMC4547048

[30] da Veiga GTS, Moriggi MR, Vettorazzi JF, Muller-Santos M, Albrecht L. Plasmodium vivax vaccine: What is the best way to go? Front Immunol. 2022;13:910236. Epub 2023/02/03. doi: 10.3389/fimmu.2022.910236 . 36726991 PMC9885200

[31] Miyazaki Y, Marin-Mogollon C, Imai T, Mendes AM, van der Laak R, Sturm A, et al. Generation of a Genetically Modified Chimeric Plasmodium falciparum Parasite Expressing Plasmodium vivax Circumsporozoite Protein for Malaria Vaccine Development. Front Cell Infect Microbiol. 2020;10:591046. Epub 2021/01/05. doi: 10.3389/fcimb.2020.591046 . 33392104 PMC7773900

[32] Salman AM, Montoya-Diaz E, West H, Lall A, Atcheson E, Lopez-Camacho C, et al. Rational development of a protective P. vivax vaccine evaluated with transgenic rodent parasite challenge models. Scientific reports. 2017;7:46482. Epub 2017/04/19. doi: 10.1038/srep46482 . 28417968 PMC5394459

[33] Lyons FMT, Gabriela M, Tham WH, Dietrich MH. Plasmodium 6-Cysteine Proteins: Functional Diversity, Transmission-Blocking Antibodies and Structural Scaffolds. Front Cell Infect Microbiol. 2022;12:945924. Epub 2022/07/29. doi: 10.3389/fcimb.2022.945924 . 35899047 PMC9309271

[34] Karbalaei M, Rezaee SA, Farsiani H. Pichia pastoris: A highly successful expression system for optimal synthesis of heterologous proteins. J Cell Physiol. 2020;235(9):5867–81. Epub 2020/02/15. doi: 10.1002/jcp.29583 . 32057111 PMC7228273

[35] Gowda DC, Miller LH. Glycosylation in malaria parasites: what do we know? Trends Parasitol. 2024;40(2):131–46. Epub 2024/01/24. doi: 10.1016/j.pt.2023.12.006 . 38262838 PMC10923157

[36] Challenger JD, Olivera Mesa D, Da DF, Yerbanga RS, Lefevre T, Cohuet A, et al. Predicting the public health impact of a malaria transmission-blocking vaccine. Nat Commun. 2021;12(1):1494. Epub 2021/03/10. doi: 10.1038/s41467-021-21775-3 . 33686061 PMC7940395

[37] Tachibana M, Sato C, Otsuki H, Sattabongkot J, Kaneko O, Torii M, et al. Plasmodium vivax gametocyte protein Pvs230 is a transmission-blocking vaccine candidate. Vaccine. 2012;30(10):1807–12. Epub 2012/01/17. doi: 10.1016/j.vaccine.2012.01.003 . 22245309

[38] Monterrubio-López GP, Delgadillo-Gutiérrez K. [Reverse vaccinology: strategy against emerging pathogens]. Revista medica del Instituto Mexicano del Seguro Social. 2021;59(3):233–41. Epub 2021/08/10. . 34370422

[39] De Groot AS, Moise L, Terry F, Gutierrez AH, Hindocha P, Richard G, et al. Better Epitope Discovery, Precision Immune Engineering, and Accelerated Vaccine Design Using Immunoinformatics Tools. Front Immunol. 2020;11:442. Epub 2020/04/23. doi: 10.3389/fimmu.2020.00442 . 32318055 PMC7154102

[40] Miura K, Tachibana M, Takashima E, Morita M, Kanoi BN, Nagaoka H, et al. Malaria transmission-blocking vaccines: wheat germ cell-free technology can accelerate vaccine development. Expert review of vaccines. 2019;18(10):1017–27. Epub 2019/10/01. doi: 10.1080/14760584.2019.1674145 . 31566026 PMC11000147

