# Supplementary figures and images for: Evaluation of transmission-blocking potential of PvPSOP25 using transgenic murine malaria parasite and clinical isolates
Source: PLoS Negl Trop Dis. 2024 Jun 12;18(6):e0012231. doi: 10.1371/journal.pntd.0012231 (PMC11168624; doi:10.1371/journal.pntd.0012231)

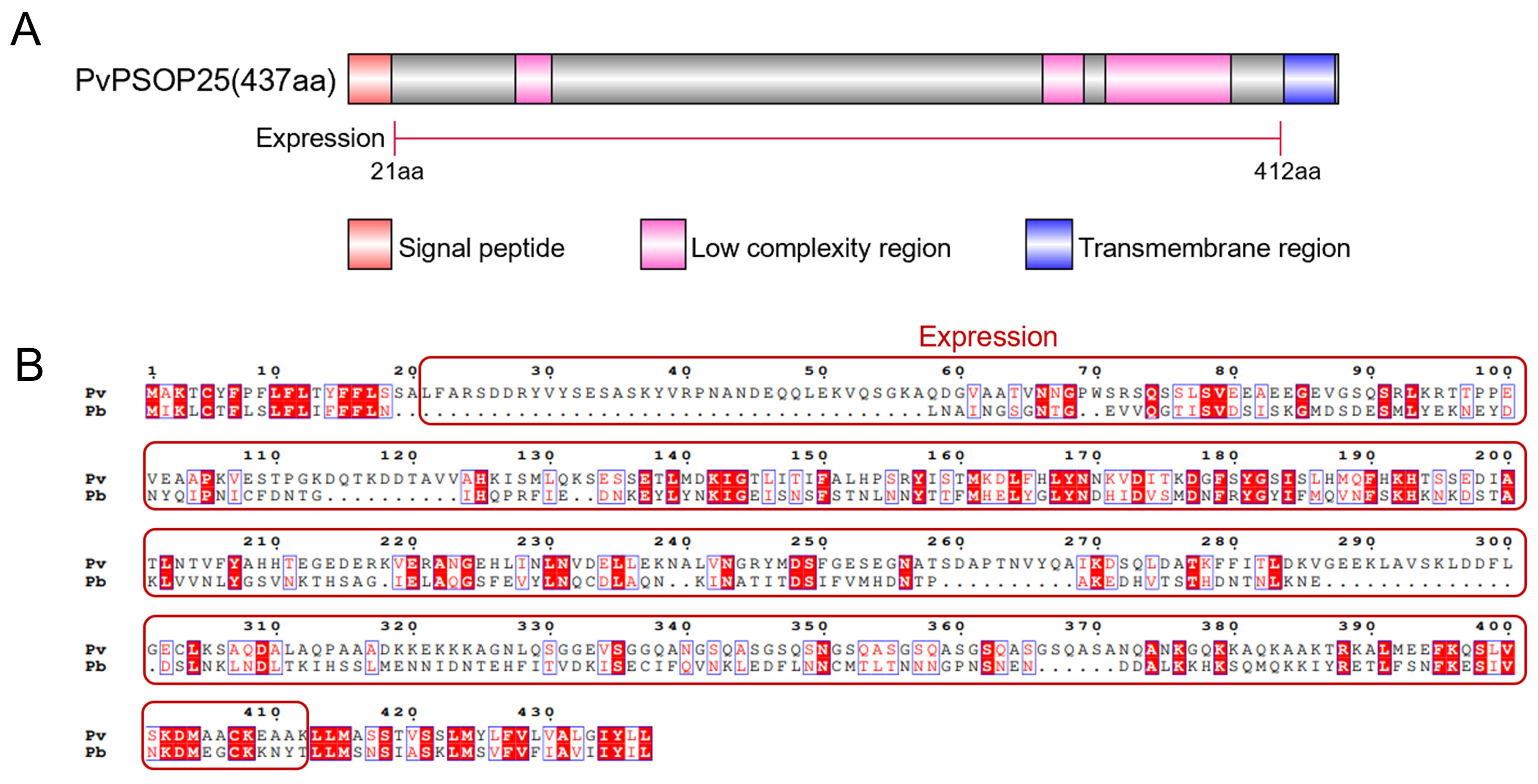

Supplement: S1 Fig — (A) PvPSOP25 contains a signal peptide (red) at the N-terminus low complexity region (pink) and transmembrane region (blue). Yeast cell expression shows amino acids 21–412. (B) Alignment of PSOP25 between P. vivax (Pv) and P. berghei (Pb). Amino acids are marked in black for identity and red for similarity. The sequence expressed in yeast cells is indicated. (TIF) [file pntd.0012231.s003.tif]

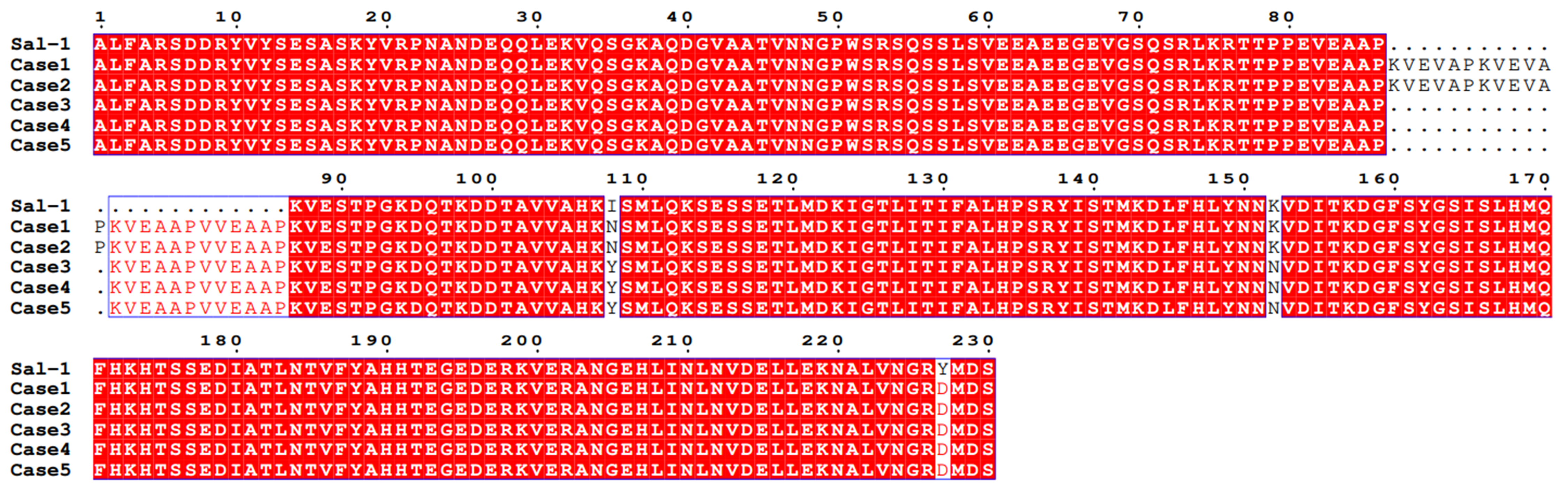

Supplement: S2 Fig — PSOP25 homologs from P. vivax reference strain (Sal-I) and five clinical samples were aligned. Identical amino acid was shadowed in red, while conserved was in white. (TIF) [file pntd.0012231.s004.tif]
